# Ehlers–Danlos hypermobility type in an adult with chronic pain and fatigue: a case study

**DOI:** 10.1002/ccr3.1046

**Published:** 2017-06-20

**Authors:** Sarah Cohen, Fred Markham

**Affiliations:** ^1^ Sidney Kimmel Medical College at Thomas Jefferson University Philadelphia Pennsylvania USA; ^2^ Thomas Jefferson University Philadelphia Pennsylvania USA

**Keywords:** Chronic fatigue, chronic pain, Ehlers–Danlos hypermobility type, joint hypermobility syndrome, joint pain

## Abstract

Ehlers–Danlos syndrome hypermobility type (EDS‐HT) is an underdiagnosed genetic connective tissue disorder that causes joint hypermobility and widespread pain. We present a patient with the chief complaint of shoulder pain, a long history of widespread joint pain, and associated comorbidities. EDS‐HT provided a unifying diagnosis and direction for management.

## Introduction

Ehlers–Danlos syndrome is a group of genetic connective tissue disorders caused by abnormal collagen formation 1. The hypermobility type is characterized by generalized joint hypermobility, as defined by the Beighton scale, and skin involvement either in the form of hyperextensibility and/or soft, velvety skin 1. Additional diagnostic criteria are recurring joint dislocations and/or subluxations, easy bruising, chronic joint pain and a positive family history, in addition to functional bowel dysfunction and autonomic dysfunction or hypotension 2. While autosomal dominant inheritance has been documented, a specific genetic mutation for EDS‐HT has not been localized; therefore, the diagnosis of EDS‐HT remains clinical 2. New data suggest that EDS‐HT is underdiagnosed: estimated frequency ranges from 1 in 20,000 to 1 in 5000 2. Thus, increased awareness of EDS‐HT diagnostic criteria in the primary care setting is of the utmost importance in two distinct areas: First, proper diagnosis can improve our grasp of the prevalence of EDS‐HT; second, early intervention for these patients can significantly decrease the impact painful symptoms and comorbidities have on patients’ lives. Our patient was a 40‐year‐old female with chronic joint pain, fatigue, headaches and GI complaints. She presented with right shoulder pain and described her shoulder as having a displaced sensation. After examination, a diagnosis of Ehlers–Danlos syndrome hypermobility type was made, changing the course of her medical care.

## Case Presentation

A 40‐year‐old Caucasian woman with history of long‐standing joint pain and chronic fatigue syndrome presented to the primary care setting for a health maintenance visit with new‐onset right shoulder pain. The pain began 1‐month prior, with a grinding quality and the sensation of joint “displacement” when she leaned on her right side. On review of systems, it was found that she has chronic headaches and joint pain, “sensitive skin,” and general itchiness.

The patient's contributing past medical history was extensive. Musculoskeletal symptoms included TMJ disorder, chronic joint and muscle pain, low back pain, and repetitive motion injuries. The patient reported that her pain began around the age of 12. Upon questioning, the patient described lifelong injuries to joints, including frequent sprained ankles, which happened seemingly with no inciting event. She reported easy bruising. She had an unconfirmed previous diagnosis of arthritis in her right thumb. Cardiovascular history included a diagnosis of orthostatic hypotension in her youth, which had never been medicated; she continues to experience dizziness and lightheadedness upon standing. Gastrointestinal history includes irritable bowel syndrome (IBS) and frequent nausea. She is being treated for deficiencies of vitamin D, for which she is taking supplements, and B12, for which she is receiving subcutaneous injections. She recently developed various chemical sensitivities but reported no medication allergies.

Unrelated previous diagnoses included diabetes mellitus type 2, hyperlipidemia, and depression. The patient's family history was noncontributory with the exception of her daughter, who is currently being tested for POTS, which has a high concurrence rate with EDS‐HT 3.

On physical examination, several abnormalities were observed. The musculoskeletal examination revealed normal strength in all extremities, with pain to palpation over the rotator cuff. Joints were examined for hypermobility using the Beighton scale criteria. The patient scored a 5/9: bilateral fifth digit passively extended to 90°, the left thumb was opposable to the forearm (examination of the right thumb was deferred secondary to prior arthritis pain), and bilateral elbow extension past 10°. She was unable to palm the floor, but expressed that she would have been able to do so easily when she was younger prior to weight gain. Integumentary examination revealed soft, velvety skin with normal extensibility. Upon drawing a tongue depressor across her forearm, an erythematous wheal appeared in under 30 seconds.

The patient was given a clinical diagnosis of Ehlers–Danlos syndrome hypermobility type. The findings of the physical examination, in particular the Beighton score of 5/9, together with the patient's history of widespread joint pain, spontaneous joint injury, and TMJ disorder support a diagnosis of EDS‐HT. Velvety, doughy skin is a major indication of the diagnosis, as well. Autonomic dysfunction, as suggested by the postural hypotension, and IBS are considered underdiagnosed comorbidities of EDS‐HT. Mast cell activation disorders, as suggested by her dermatographia and chemical sensitivities, are being examined for possible connection to EDS‐HT 4.

The major diagnostic challenge was in recognizing the underlying diagnosis that connected her wide and complex history. As she suffered from several more easily diagnosed conditions – diabetes, arthritis, and depression – as well as being overweight, her joint pain had largely been dismissed. Fortunately, on this occasion, her description of shoulder instability prompted an examination for joint hypermobility. With that unifying condition, the rest of the symptoms fell into place.

The patient was referred to Cardiology for an echo to rule out vascular involvement, in particular aortic root dilation. For management, she was referred to Maxillofacial and Oral Surgery for TMJ pain control and Physical Medicine and Rehabilitation for joint pain control and physical assessment. Physical therapy and pharmacotherapy may be recommended in the future.

## Discussion

Ehlers–Danlos syndrome hypermobility type is considered an underdiagnosed heritable connective tissue disorder (HCTD), explaining the delay in diagnosis for our patient 5. Presentation of EDS‐HT can be vague, with widespread symptoms that present a challenging picture to unite into one diagnosis, making awareness of these nuances all the more important.

Genetic testing has limited value; there is no genetic or biochemical test for EDS‐HT, as the etiology is unknown and likely heterogeneic. Genetic testing is primarily important when concern for vascular involvement exists, because vascular type (vEDS) predisposes patients to spontaneous rupture of hollow organs, causing significant morbidity and mortality. *COL3A1* is dysfunctional and/or deficient in vEDS; however, failure to find a pathogenic variant does not entirely rule out the vEDS 2. The differential diagnosis for EDS types can be seen in Table 1.

**Table 1:** Criteria for the historical EDS types and underlying molecular defect 10

Type	Distinctive features	Inheritance pattern	Gene (enzyme)
Classic (EDS I, EDS II)	Skin hyperextensibility Widened, atrophic scars Soft, velvety skin Skin and soft tissue fragility Generalized joint laxity and pain	Autosomal dominant	*COL5A1*,* COL5A2*
Hypermobility (EDS III)	Generalized joint laxity and pain Smooth, velvety skin Recurring joint dislocations	Autosomal dominant	Unknown
Vascular (EDS IV)	Thin, translucent, fragile skin Rupture or fragility of hollow organs Joint laxity in small joints Family history of sudden death	Autosomal dominant	*COL3A1*
Kyphoscoliosis (EDS VI)	Scoliosis at birth Scleral fragility and rupture of ocular globe Severe skin manifestations	Autosomal recessive	*PLOD1* (lysyl hydroxylase)
Arthrochalasia (EDS VIIA, EDS VIIB)	Congenital hip dislocation Skin hyperextensibility and fragility	Autosomal dominant	*COL1A1*,* COL1A2*
Dermatosparaxis (EDS VIIC)	Skin fragility Sagging, soft, doughy skin Premature rupture of membranes	Autosomal recessive	*ADAMTS2* (procollagen peptidase)

The mainstay of Ehlers–Danlos interventions is lifestyle modifications and pain management. As described elsewhere, physical therapy is complementary for strengthening core muscles and supporting joints with low‐impact exercises 6. Similarly, Physical Medicine and Rehabilitation, to develop appropriate pain management interventions, and Orthopedics can provide custom braces for additional joint support 5. Splinting of major joints as well as fingers can help to prevent joint injury and preempt pain symptoms 5. Nerve blocks, and lidocaine and steroid injections are sometimes recommended for pain management as an alternative to systemic pain medication, but show limited efficacy 5. Additionally, massage therapy, heat and cold therapy, and splinting for joint support can all offer significant improvements.

Pharmacologic intervention requires further study. Some patients find relief from the use of opioid pain medications, while others make use of NSAIDs and acetaminophen during painful episodes 5. Pharmacotherapy targeting neuropathic pain such as pregabalin (Lyrica^©^, Pfizer, New York, NY, USA), gabapentin (Neurontin^©^, Pfizer, New York, NY, USA), or amitriptyline have shown some efficacy, suggesting an overlap with neuropathic pain syndromes or fibromyalgia 7, 8. Management by a rheumatologist can be beneficial, not only for joint pain management, but also because it was recently shown that EDS‐HT is associated with several inflammatory and noninflammatory rheumatologic disorders, including rheumatoid arthritis and fibromyalgia 9.

Surgery should be avoided to the extent possible, as many EDS‐HT patients experienced slower healing and abnormal scarring, as well as abnormal responses to anesthesia 5.

Psychological counseling should be made available to all patients both for learning pain management coping techniques and for emotional support. EDS often has comorbid anxiety and depression 5. Due to the unrecognized nature of EDS, patients often are made to feel discredited and isolated, and should be offered therapeutic support.

## Conclusion

This report details the unusual presentation of a patient who has suffered from joint pain from EDS‐HT and associated comorbidities. We have outlined the plan for treatment and symptom relief for her. The patient expressed, even before interventions began, that finally having a diagnosis changed everything for her. It should be stressed that proper recognition of her true medical condition provided enormous relief, and this should not be overlooked when treating patients.

## Authorship

SC: prepared manuscript. FM: guided the author in writing the manuscript and proofread the final manuscript.

## Conflict of Interest

None declared.
